# Histopathological changes of the Eustachian tube mucosa following balloon Eustachian tuboplasty in children: insights from standard endovascular balloon application

**DOI:** 10.1038/s41598-026-57118-9

**Published:** 2026-07-21

**Authors:** Abdelrahman Mostafa Hassan, Hani Farouk Elgarem, Eman Sheta, Yasser Gaber Sheweal, Yehia Mohammed Ashry

**Affiliations:** 1 Faculty of Medicine, Alexandria University, Alexandria, Egypt; 2 Faculty of Medicine, Suez Canal University, Ismailia, Egypt

**Keywords:** Balloon dilation, Eustachian tube dysfunction, Endovascular balloon, Histopathology, Diseases, Medical research

## Abstract

Balloon Eustachian tuboplasty (BET) has emerged as an established intervention for obstructive Eustachian tube dysfunction; however, the high cost of proprietary balloon systems limits widespread accessibility. Standard endovascular balloons represent a low-cost alternative, yet their tissue-level effects remain insufficiently characterized, particularly in pediatric populations. This study aimed to evaluate histopathological changes of the Eustachian tube (ET) mucosa before and after BET using a standard endovascular balloon. Twenty-two Eustachian tubes (ETs) from eleven children with bilateral obstructive dysfunction refractory to medical and prior surgical management underwent BET. Mucosal biopsies were obtained immediately before and after dilation in all cases. In addition, follow-up biopsies at 16 weeks were obtained from two ETs in a single patient to explore short-term histopathological changes. Specimens were independently analyzed by two blinded pathologists using light microscopy, focusing on epithelial integrity, inflammatory infiltration, lymphoid tissue architecture, and fibrosis. Pre-dilation specimens demonstrated epithelial inflammation with dense submucosal lymphocytic infiltration. Immediately following dilation, mucosal thinning, partial epithelial disruption, and compression of lymphoid tissue were observed, while the basal epithelial layer remained largely preserved. At 16 weeks, limited follow-up specimens demonstrated partial re-epithelialization with restoration toward pseudostratified ciliated columnar epithelium and reduction in inflammatory infiltrates, without histological evidence of tissue necrosis or marked fibrosis in the sampled specimens. BET using a standard endovascular balloon was associated with immediate epithelial and submucosal injury-related histopathological alterations while preserving the basal epithelial layer. Limited follow-up histological observations from a single case demonstrated partial re-epithelialization with reduced inflammatory infiltrates. However, these findings should be considered preliminary and do not permit definitive conclusions regarding reparative response, clinical tolerability, safety, feasibility, or long-term tissue remodeling, which require validation in larger prospective longitudinal studies.

**Supplementary Information:**

The online version contains supplementary material available at https://doi.org/10.1038/s41598-026-57118-9.

## Introduction

The ET is the anatomical channel connecting the middle ear to the nasopharynx. Normal ET function is essential for maintaining middle ear aeration and pressure equilibrium with the external environment. In addition to pressure equalization, the ET facilitates clearance of middle ear secretions and helps prevent reflux of nasopharyngeal contents and sound transmission into the middle ear^1,2^.

When the ET fails to open adequately, most commonly because of inflammatory or mucosal alterations within its lumen triggered by infection or irritants, Eustachian tube dysfunction (ETD) develops. Impaired tubal ventilation results in disequilibrium between middle ear and nasopharyngeal pressures as atmospheric gases are absorbed through the middle ear mucosa. Persistent dilatory dysfunction may manifest clinically as aural fullness, popping sensations, hearing loss, tinnitus, autophony, otalgia, and occasionally imbalance^3^.

BET, targeting the cartilaginous portion of the ET, has emerged as a therapeutic option for patients with chronic dilatory dysfunction who remain symptomatic despite conventional medical therapy. Reported indications include otitis media with effusion, persistent negative middle ear pressure, non-adhesive tympanic membrane atelectasis, and difficulty tolerating ambient pressure changes such as those encountered during air travel or diving^4–8^. Although favorable clinical outcomes have been reported following BET, the biological mechanisms underlying functional improvement remain incompletely understood. Our previous clinical study demonstrated significant improvement in tympanometric outcomes following balloon dilation using a standard endovascular balloon catheter^9^, underscoring the need to better characterize the associated tissue-level changes.

Although BET has demonstrated beneficial clinical outcomes in selected patients, a subset derives limited or no clinical benefit. The absence of standardized approaches for managing BET failure may partly reflect limited histopathological data describing mucosal and submucosal tissue responses after dilation. Previous reports have documented microscopic alterations, including mucosal disruption and changes in inflammatory tissue characteristics, following BET^10,11^. Kivekäs et al. described immediate epithelial shearing, mucosal thinning, and submucosal crush injury with lymphocytic infiltration, followed postoperatively by restoration of pseudostratified columnar epithelium and replacement of inflammatory infiltrates with fibrous tissue^11^.

Despite these insights, histopathological evaluation of the ET after BET has been reported in only a limited number of experimental studies and a single human study using dedicated balloon systems. Consequently, direct histopathological evidence describing tissue-level alterations following BET in clinical settings remains scarce. Moreover, the high cost and limited availability of proprietary balloon devices represent substantial barriers, particularly in resource-constrained environments. Standard endovascular balloons may offer a more accessible and cost-effective alternative; however, their histopathological effects remain insufficiently characterized.

Therefore, the present study aimed to evaluate histopathological changes of the ET mucosa before and after BET in a pediatric population using a standard endovascular balloon catheter, with particular emphasis on epithelial integrity, inflammatory response, and submucosal structural alterations.

## Materials and methods

### Ethical approval and patient selection

The study was approved by the local institutional ethics committee (IRB approval No. 00012098). All procedures were conducted in accordance with the ethical standards of the responsible committee and the Declaration of Helsinki. Written informed consent was obtained from all parents or legal guardians prior to enrollment, including specific consent for intraoperative ET mucosal biopsy.

Patients aged 7–18 years with chronic dilatory ETD were prospectively enrolled. Inclusion criteria consisted of bilateral persistent otitis media with effusion or non-adherent tympanic membrane atelectasis. All patients had failed appropriate medical management, including at least six weeks of intranasal corticosteroid therapy, and had previously undergone one or more tympanostomy tube insertions that resulted only in temporary clinical improvement until tube extrusion followed by symptom recurrence. Long-term adjunctive medical therapies, such as antihistamines and proton pump inhibitors, were continued when clinically indicated.

### BET and biopsy technique

All procedures were performed under general anesthesia using endoscopic guidance, targeting the cartilaginous portion of the ET. A combined transnasal–transoral endoscopic approach was used in all cases. A 45° rigid nasal endoscope was introduced transnasally to visualize the ET orifice, while a curved metal suction catheter was introduced transorally and used as a guiding instrument. The endovascular balloon catheter was advanced under direct endoscopic visualization along the suction catheter using a Seldinger-like technique to facilitate controlled and precise positioning within the cartilaginous ET.

Mucosal biopsies were obtained transorally from the posterior cushion of the ET, specifically from the superior aspect within the tubal lumen. Biopsies were collected immediately before balloon dilation and repeated directly after dilation using Giraffe forceps under endoscopic guidance. Post-dilation biopsies were intentionally obtained from an adjacent, previously unbiopsied area of the posterior cushion to minimize potential confounding related to sequential sampling from the same site. To ensure anatomical consistency and minimize sampling variability, all biopsies and dilation procedures were performed by the same surgeon using a standardized surgical technique.

Balloon dilation was performed using a 6 mm × 20 mm standard endovascular balloon catheter (Sterling™ Monorail™, Boston Scientific), inflated to 10–12 atmospheres and maintained for 1.5 min. The 1.5-minute dilation duration was selected to balance procedural efficacy and safety in the pediatric population, consistent with previous clinical observations recommending shorter dilation durations in patients younger than 18 years of age^12^. Balloon advancement was halted immediately if resistance was encountered, and care was taken not to advance the catheter beyond the visible proximal marker to minimize the risk of mucosal injury or inadvertent entry into the osseous ET. The balloon was then deflated and withdrawn, and all biopsy specimens were immediately submitted for histopathological processing.

As all procedures were performed using the same standardized combined transnasal–transoral technique, variability related to differences in directional pressure application was minimized.

In one patient, additional follow-up biopsies were obtained from both ETs at 16 weeks postoperatively during an unrelated nasal surgical procedure performed under general anesthesia.

### Histopathological assessment

All ET mucosal biopsies were fixed immediately in 10% neutral buffered formalin. Specimens were placed on edge in plastic cassettes to preserve orientation, processed through ascending grades of alcohol, cleared in xylene, and embedded in paraffin wax. Sections of 5 μm thickness were cut using a semi-automated microtome, mounted on glass slides, and stained with hematoxylin and eosin (H&E) for light microscopic examination.

Histopathological evaluation was conducted according to previously described criteria^11^. The following parameters were assessed in both pre- and post-dilation specimens:

### Surface mucosa

#### Epithelial quality:

scored from 0 to 3.

(0 = totally ulcerated surface epithelium; 1 = fair; 2 = good; 3 = excellent intact epithelium)

#### Cilia:

scored as 0 = absent, 1 = fair, 2 = good (assessed at ×400 magnification).

#### Squamous metaplasia:

0 = absent, 1 = present.

#### Intraepithelial inflammation:

0 = absent, 1 = present.

#### Basal cell integrity:

scored from 0 to 3.

(0 = completely lost; 3 = intact and of good quality), recognizing the basal epithelial layer as the regenerative epithelial compartment.

### Submucosa

#### Submucosal inflammation:

scored as 0 = absent, 1 = mild, 2 = moderate, 3 = severe.

#### Lymphoid follicles:

0 = absent, 1 = present.

#### Fibrosis:

scored as 0 = none, 1 = mild, 2 = moderate, 3 = severe.

#### Submucosal glands:

seromucinous acini assessed as.

0 = not sampled, 1 = fair (degeneration, dilatation, or rupture), 2 = good, 3 = excellent.

#### Crushing artifact:

assessed in lymphoid tissue as.

0 = absent or minimal crushing; 1 = moderate to marked crushing, characterized by basophilic streaks, loss of cellular detail, and nuclear smudging.

All biopsies were initially evaluated in the routine histopathology laboratory by a pathologist blinded to clinical data and biopsy timing. Slides and paraffin blocks were subsequently re-assessed independently by a consultant pathologist, also blinded to clinical information and biopsy timing.

### Postoperative follow-up and tympanometric assessment

All patients were followed for 6 months after the procedure. Tympanometric evaluation was performed at the 6-month follow-up visit. Tympanograms were classified as type A, indicating normal middle ear pressure and compliance; type B, indicating a flat tympanogram suggestive of persistent middle ear effusion; and type C, indicating negative middle ear pressure suggestive of residual ETD.

Based on the 6-month postoperative tympanometric findings, treated ears were categorized into two groups. Group I included ears demonstrating objective tympanometric improvement, whereas Group II included ears that did not meet the predefined improvement criterion. Objective tympanometric improvement was defined as conversion from type B to type A or C, or from type C to type A at the 6-month follow-up evaluation.

Detailed audiological and symptom-based clinical outcomes were evaluated separately in a previously published clinical study^9^ and are therefore only briefly referenced in the present histopathological investigation.

### Statistical analysis

Data were entered into a computer and analyzed using IBM SPSS Statistics for Windows, version 27.0 (IBM Corp., Armonk, NY, USA; released 2020). Continuous variables were summarized as mean ± standard deviation (SD) or median with interquartile range (IQR), according to data distribution. Categorical variables were presented as frequencies and percentages. Normality of continuous variables was assessed using the Shapiro–Wilk test.

Because both ETs from the same patient were included in the analysis, statistical methods accounting for within-patient clustering and the non-independence of bilateral observations were applied where appropriate. Generalized estimating equation (GEE) models were used to evaluate changes in mucosal and submucosal histopathological parameters before and after balloon dilation, with patient identifier specified as the clustering variable and time point (pre- versus post-dilation) included as the main effect. Regression coefficients (β), Wald χ² statistics, odds ratios (ORs), and corresponding 95% confidence intervals (CIs) were calculated.

Descriptive paired comparisons between pre- and post-dilation histopathological findings were additionally evaluated using the marginal homogeneity test or McNemar’s test for paired categorical variables, as appropriate. Ordinal or non-normally distributed paired variables were analyzed using the Wilcoxon signed-rank test. Effect sizes were reported using Cramer’s V, phi coefficient (φ), and Wilcoxon effect size (r), where appropriate.

Inter-observer agreement between the two blinded pathologists was assessed using intraclass correlation coefficients (ICCs), based on a two-way mixed-effects model with consistency definition, and Cohen’s weighted kappa statistics with quadratic weighting, together with corresponding 95% confidence intervals. Agreement strength was interpreted according to previously published recommendations. A two-sided p-value < 0.05 was considered statistically significant.

## Results

BET was performed on 22 ETs in 11 pediatric patients (6 males and 5 females) between April 2024 and April 2025. The mean patient age was 10 years (range, 7–14 years). At 6 months postoperatively, tympanometric improvement to type A or C was observed in 80% of treated ears, whereas the remaining ears continued to demonstrate persistent type B tympanograms at follow-up.

No complications related to biopsy sampling were observed. All biopsy sites healed uneventfully with minimal scarring. Intraoperative bleeding was limited and did not require cautery or topical vasoconstrictors.

Inter-observer reliability analysis demonstrated good-to-excellent agreement between both blinded pathologists across all evaluated histopathological parameters (ICC: 0.901–1.000; weighted κ: 0.805–1.000). (Supplementary Table S1)

### Pre-dilation histopathological findings

Histopathological alterations of the ET mucosa were identified in all pre-dilation biopsy specimens. Ulceration of the surface respiratory epithelium was observed in four specimens (18.2%). The remaining specimens demonstrated fair epithelial quality in five cases (22.7%), good quality in ten cases (45.5%), and excellent epithelial integrity in three cases (13.6%). Squamous metaplasia was identified in two specimens (9.1%). Cilia were absent in eight cases (36.4%), partially preserved in ten cases (45.5%), and well preserved in four cases (18.2%).

Assessment of the basal epithelial layer revealed generally preserved integrity, graded as excellent in ten specimens (45.5%), good in seven (31.8%), and fair in five (22.7%).

**Fig. 1:**
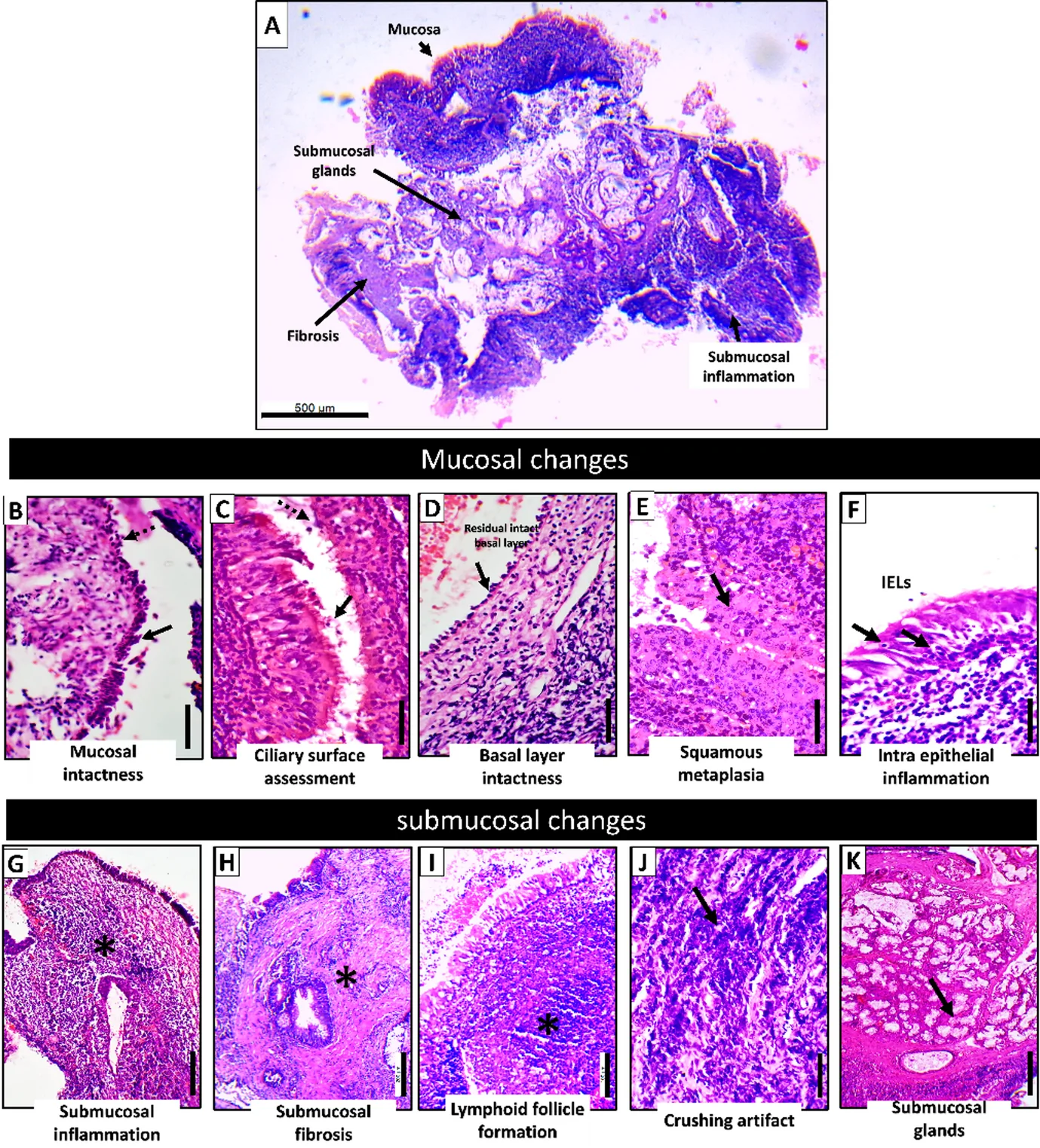
Histopathological assessment of H&E-stained sections of ET biopsies.** (A)** Low-power overview demonstrating the anatomical components of the biopsy specimen, including mucosa, submucosal glands, inflammatory infiltrates, and fibrosis (×40, scale bar = 500 μm). Representative mucosal histopathological findings:** (B)** Mucosal intactness assessment: Surface respiratory epithelium demonstrating focal epithelial ulceration (dashed arrow) adjacent to preserved respiratory epithelium (arrow) (×100, scale bar = 200 μm). **(C)** Assessment of ciliary surface integrity. Arrow points at intact ciliary border. (×400, scale bar = 50 μm). **(D)** Basal layer intactness: Residual intact basal epithelial layer beneath an area of epithelial ulceration (arrow) (×200, scale bar = 100 μm). **(E)** Squamous metaplasia with replacement of respiratory epithelium by squamous epithelium (arrow) (×400, scale bar = 50 μm). **(F)** Intraepithelial inflammation: characterized by intraepithelial lymphocytes (IELs) (arrows) (×400, scale bar = 50 μm). Representative submucosal histopathological findings:** (G)** Submucosal inflammatory infiltrates (*) (×100, scale bar = 200 μm). **(H)** Submucosal fibrosis characterized by dense collagen deposition (*) (×100, scale bar = 200 μm).**(I)** Lymphoid follicle formation (*) (×200, scale bar = 100 μm).**(J)** Crushing artifact characterized by basophilic streaking with loss of cellular detail and nuclear smudging (arrow) (×400, scale bar = 50 μm). **(K)** Submucosal seromucinous glands identified within the sampled depth of the biopsy specimen (arrow) (×100, scale bar = 200 μm).

Submucosal inflammation was present in all pre-dilation specimens, with variable severity. Lymphoid follicles were identified in four specimens (18.2%) and were frequently associated with intraepithelial lymphocytic infiltration, which was present in 50% of cases. Fibrotic changes were observed in the majority of specimens, graded as moderate in 16 cases (72.7%), severe in four cases (18.2%), and mild in two cases (9.1%). Submucosal glands were not identified within the sampled depth of the biopsy in 12 specimens (54.5%), while excellent glandular morphology was observed in eight specimens (36.4%); fair and good preservation were each noted in one specimen (4.5%). (Fig. 1)

### Post-dilation histopathological findings

Biopsies obtained immediately after balloon dilation demonstrated acute mechanical tissue alterations. GEE analysis demonstrated significant post-dilation alterations in epithelial quality (OR 0.089, 95% CI 0.026–0.313, *p* < 0.001), ciliary status (OR 0.186, 95% CI 0.079–0.435, *p* < 0.001), and intraepithelial inflammation (OR 0.158, 95% CI 0.049–0.509, *p* = 0.002). Crushing artifact was also significantly more frequent following dilation (OR 26.667, 95% CI 4.181–170.091, *p* < 0.001). (Tables 1 and 2)

**Table 1:** Generalized estimating equation (GEE) analysis of mucosal and submucosal histopathological parameters before and after balloon Eustachian tuboplasty.

		Effect	Wald χ²	df	p-value
Mucosal histopathologic findings	Epithelium quality	Time	14.307	1	< 0.001^*^
	Cilia	Time	15.076	1	< 0.001^*^
	Squamous metaplasia	Time	0.000	1	1.000
	Intraepithelial inflammation	Time	9.537	1	0.002^*^
	Basal cell integrity	Time	1.422	1	0.233
Submucosal histologic parameters	Submucosal inflammation	Time	0.549	1	0.459
	Lymphoid follicles	Time	2.861	1	0.091
	Quantity of fibrosis	Time	1.122	1	0.289
	Gland	Time	2.625	1	0.105
	Crushing artifact	Time	12.062	1	< 0.001^*^

GEE: generalized estimating equation; df: degrees of freedom.

*Statistically significant at *p* ≤ 0.05.

**Table 2:** Parameter estimates from generalized estimating equation (GEE) analysis of histopathological parameters before and after balloon Eustachian tuboplasty.

Variable		Estimate β	Std. Error	Wald χ²	*p*-value	Odds ratio (OR)	95% CI for OR
Epithelium quality	Time [after]	-2.414	0.638	14.307	< 0.001^*^	0.089	0.026–0.313
Cilia	Time [after]	-1.683	0.433	15.076	< 0.001^*^	0.186	0.079–0.435
Squamous metaplasia	Time [after]	0.000	1.100	0.000	1.000	1.000	0.116–8.636
Intraepithelial inflammation	Time [after]	-1.846	0.598	9.537	0.002	0.158	0.049–0.509
Basal cell integrity	Time [after]	-0.514	0.431	1.422	0.233	0.598	0.257–1.392
Submucosal inflammation	Time [after]	-0.302	0.407	0.549	0.459	0.739	0.333–1.643
Lymphoid follicles	Time [after]	-1.540	0.911	2.861	0.091	0.214	0.036–1.277
Quantity of fibrosis	Time [after]	-0.701	0.662	1.122	0.289	0.496	0.136–1.814
Submucosal Gland	Time [after]	0.961	0.593	2.625	0.105	2.616	0.817–8.369
Crushing artifact	Time [after]	3.283	0.945	12.062	< 0.001^*^	26.667	4.181–170.091

GEE: generalized estimating equation; OR: odds ratio; CI: confidence interval; β: regression coefficient.

*Statistically significant at *p* ≤ 0.05.

**Fig. 2:**
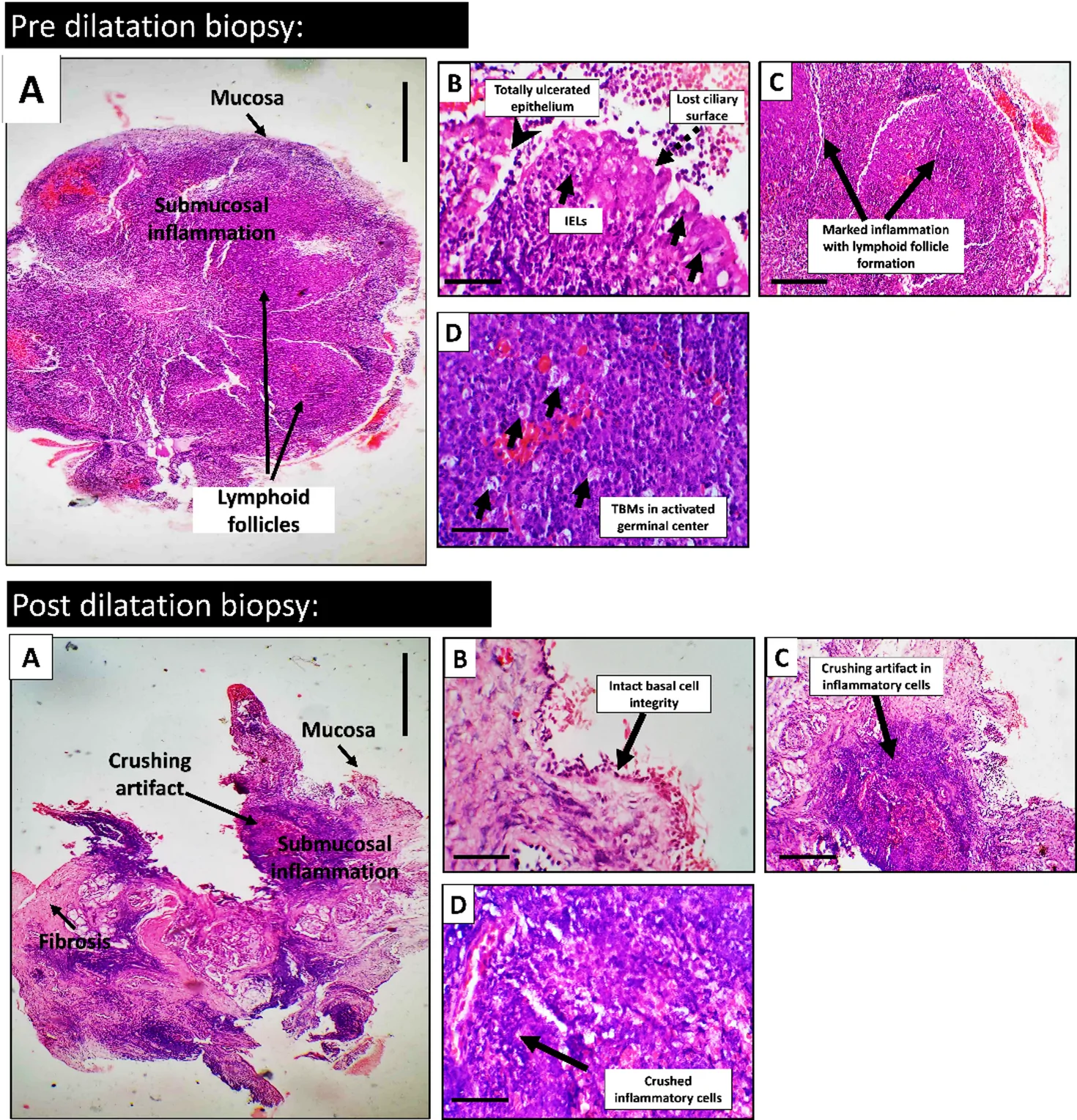
Histopathological assessment of H&E-stained sections obtained before and immediately after balloon dilation of the ET. Pre-dilation biopsy:** (A)** Low-power view demonstrating mucosa (arrow) and submucosa expanded by severe chronic inflammatory infiltrates with lymphoid follicle formation; no seromucinous acini are identified within the sampled depth (×40, scale bar = 500 μm).**(B)** High-power view demonstrating focal epithelial ulceration (arrowhead), loss of ciliary surface (dashed arrows), and numerous intraepithelial lymphocytes (IELs, arrows) (×400, scale bar = 50 μm). **(C)** Marked chronic inflammatory infiltrates with lymphoid follicle formation (arrows) (×100, scale bar = 200 μm). **(D)** Activated germinal centers with tingible body macrophages (TBMs) (arrows) (×400, scale bar = 50 μm). Post-dilation biopsy:** (A)** Low-power view demonstrating preserved overall anatomical architecture with mucosa (arrow) and submucosa expanded by chronic inflammatory infiltrates showing crushing artifact; areas of fibrosis are also present (×40, scale bar = 500 μm). **(B)** High-power view demonstrating ulcerated respiratory epithelium with preservation of the basal epithelial layer (arrow) (×400, scale bar = 50 μm). **(C)** Submucosal inflammatory infiltrates demonstrating crushing artifact (arrow) (×100, scale bar = 200 μm). **(D)** High-power view demonstrating crushed inflammatory cells (arrow) with elongated smudged nuclei (×400, scale bar = 50 μm).

Surface epithelial ulceration increased following dilation and was identified in 16 post-dilation specimens (72.7%) compared with four specimens (18.2%) pre-dilation. Similarly, absent cilia were observed in 16 post-dilation specimens (72.7%) compared with eight specimens (36.4%) before dilation. Despite these epithelial alterations, basal epithelial layer integrity remained largely preserved, with no statistically significant difference between pre- and post-dilation specimens after cluster-adjusted analysis (*p* = 0.233). (Fig. 2)

No statistically significant post-dilation alterations were observed in squamous metaplasia (*p* = 1.000), submucosal inflammation severity (*p* = 0.459), presence of lymphoid follicles (*p* = 0.091), quantity of fibrosis (*p* = 0.289), or submucosal glandular features (*p* = 0.105). (Table 2)

Descriptive paired distributions of the histopathological parameters before and after dilation are presented in Supplementary Tables S2 and S3. Descriptive subgroup analyses according to the 6-month tympanometric outcome are presented in Supplementary Tables S4 and S5. Ears demonstrating postoperative tympanometric improvement (Group I) showed some numerical differences compared with non-improved ears (Group II), particularly regarding basal epithelial layer preservation. Improved ears showed a descriptively higher proportion of excellent basal epithelial integrity both before and immediately after dilation compared with non-improved ears. However, these subgroup observations did not demonstrate statistically significant clinicopathological associations and should therefore be interpreted cautiously, given the limited sample size and exploratory nature of the analysis.

### Follow-up histopathological findings

Limited follow-up histopathological assessment was available from one patient who underwent additional bilateral ET biopsies at 16 weeks postoperatively during an unrelated nasal surgical procedure.

**Fig. 3:**
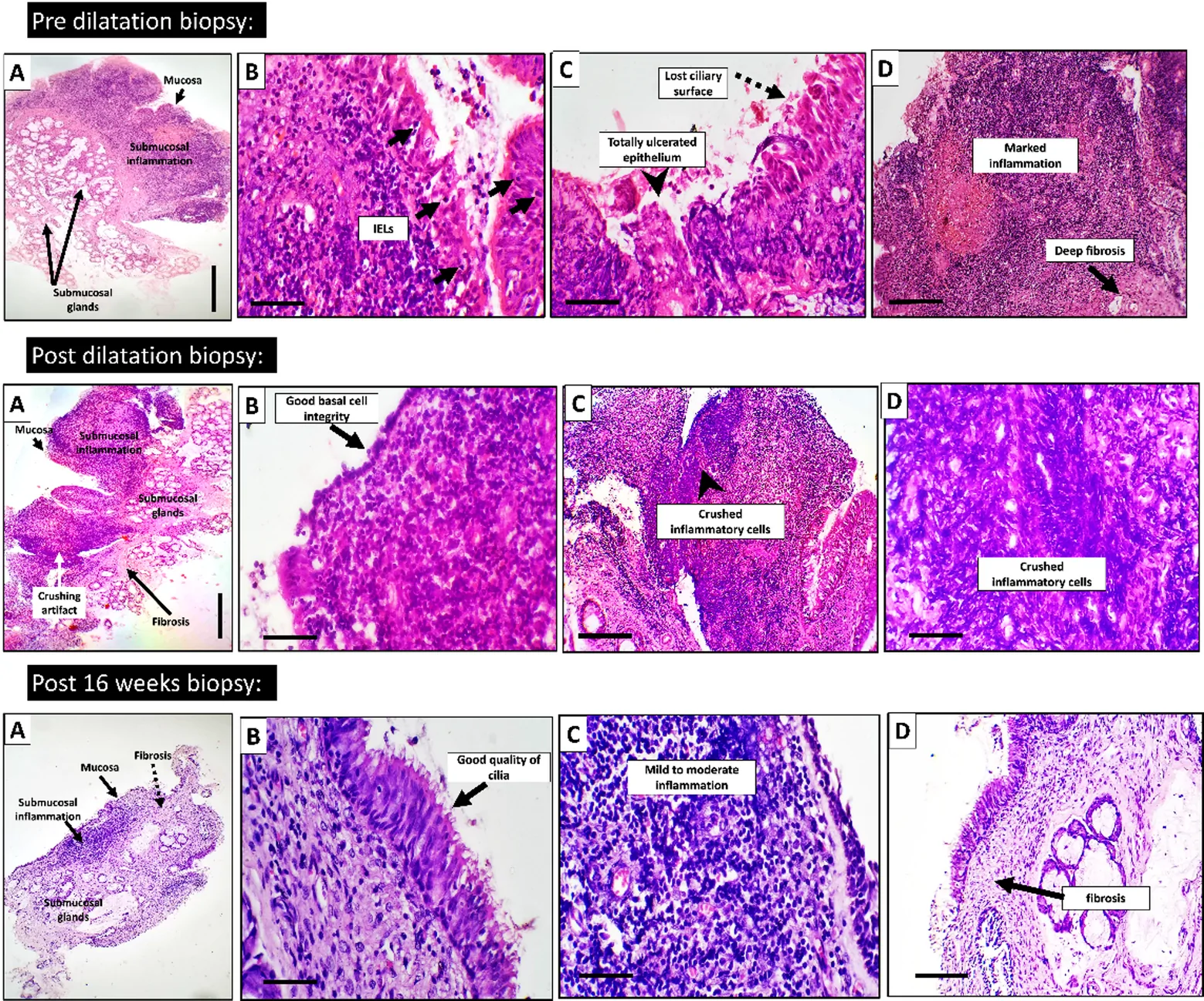
Comparative histopathological assessment of H&E-stained sections obtained before balloon dilation, immediately after dilation, and at 16-week follow-up. Pre-dilation biopsy:** (A)** Low-power view demonstrating mucosa, submucosal glands, and submucosa expanded by chronic inflammatory infiltrates (×40, scale bar = 500 μm). **(B)** High-power view demonstrating numerous intraepithelial lymphocytes (IELs) within the respiratory epithelium (arrows) (×400, scale bar = 50 μm). **(C)** Areas of epithelial ulceration (arrowhead) with loss of ciliary surface (dashed arrows) (×400, scale bar = 50 μm). **(D)** Marked chronic inflammatory infiltrates associated with deep fibrosis (arrow) (×100, scale bar = 200 μm). Post-dilation biopsy: **(A)** Low-power view demonstrating mucosa, submucosal glands, fibrosis, and crushing artifact within areas of chronic inflammatory infiltrates (×40, scale bar = 500 μm). **(B)** High-power view demonstrating preservation of the basal epithelial layer with good integrity (arrow) (×400, scale bar = 50 μm). **(C)** Submucosal inflammatory infiltrates demonstrating crushing artifact (arrowhead) (×100, scale bar = 200 μm). **(D)** High-power view demonstrating crushed inflammatory cells with smudged nuclei (×400, scale bar = 50 μm). Sixteen-week follow-up biopsy:** (A)** Low-power view demonstrating reduced submucosal inflammatory infiltrates, preserved submucosal glands (arrows), and mild fibrosis (dashed arrow) (×40, scale bar = 500 μm). **(B)** High-power view demonstrating intact respiratory epithelium with preserved ciliary surface of good quality (arrow) (×400, scale bar = 50 μm). **(C)** Mild-to-moderate residual submucosal inflammatory infiltrates (×100, scale bar = 200 μm). **(D)** Mild submucosal fibrosis (arrow) (×100, scale bar = 200 μm).

Histological evaluation at 16 weeks demonstrated restoration toward ciliated pseudostratified columnar epithelium with an intact basal epithelial layer. Compared with immediate post-dilation specimens, less prominent inflammatory infiltrates were observed. In one ET, a lymphoid follicle that had appeared flattened and crushed in the immediate post-dilation specimen appeared to be associated with a thinner fibrous layer. Submucosal glands appeared preserved, with no overt histological features suggestive of glandular injury related to balloon dilation. (Fig. 3)

## Discussion

The pre-dilation histopathological findings in this study reflect the clinical phenotype of chronic inflammatory disease affecting the ET orifice and lumen. Surface epithelial disruption, ciliary loss, and inflammatory cell infiltration were consistently observed, in some cases extending through the full epithelial thickness. Submucosal inflammation was prominent in most specimens, and lymphoid follicle formation was identified in a subset of cases. Clinically, cobblestoning of the nasopharynx was frequently observed and corresponded histologically to adenoid-like lymphoid tissue within the tubal lumen. These findings are consistent with a chronically inflamed mucosal environment and suggest contributions from persistent inflammatory drivers such as allergic rhinitis, chronic rhinosinusitis, or biofilm-associated disease processes. Christov and Gluth reported comparable inflammatory and lymphoid changes at the protympanic end of the ET in patients with chronic otitis media with effusion^13^.

Biopsies obtained immediately after balloon dilation demonstrated characteristic acute mechanical alterations consistent with previously reported effects of BET. Lymphoid tissue frequently exhibited crushing artifacts with flattening and distortion, while the basal epithelial layer remained preserved. Surface epithelial integrity declined, with ulceration and ciliary loss observed in the majority of specimens. These findings closely mirror those described by Poe et al., who reported that balloon dilation induces superficial mucosal injury while relatively preserving deeper structural elements of the ET mucosa^8^. The preservation of the basal epithelial layer observed in the present cohort may reflect preservation of the regenerative epithelial compartment following dilation and a potential for subsequent epithelial recovery.

The inflation pressures used in the present study were selected within the pressure ranges commonly employed by commercially available BET systems, several of which utilize non-compliant balloon technology^14^. Previous pediatric BET studies and contemporary clinical reports have similarly utilized inflation pressures of approximately 10–12 atm with dilation durations ranging from 1 to 2 min, supporting the clinical applicability of the current protocol in pediatric patients^15–21^. In the present cohort, gradual inflation to 10–12 atm for approximately 1.5 min was intentionally adopted to balance adequate luminal expansion while minimizing excessive mechanical trauma or over-dilation.

Biomechanical investigations utilizing the Sterling Monorail balloon as a structurally comparable alternative to dedicated BET devices demonstrated that balloon dilation may induce controlled mechanical tissue deformation, including mucosal tearing and focal cartilage cracking, findings considered part of the mechanical expansion process associated with ET dilation rather than uncontrolled tissue disruption^22^. These investigations further demonstrated that the majority of tissue deformation occurs at relatively lower inflation pressures, with comparatively limited additional deformation at higher pressures because of the non-compliant characteristics of the balloon system^22^.

Importantly, the histopathological findings of the current study should be interpreted cautiously. Although preservation of the basal epithelial layer was observed in most specimens, and limited postoperative histological observations from a single patient demonstrated partial re-epithelialization with reduced inflammatory infiltrates, these findings remain preliminary and descriptive. Therefore, they do not permit definitive conclusions regarding biological remodeling, reparative tissue response, long-term mucosal recovery, or clinical tolerability following dilation. Larger prospective pediatric studies incorporating longitudinal histopathological assessment are required to further clarify these processes.

Limited postoperative histopathological assessment at 16 weeks from one pediatric patient demonstrated partial re-epithelialization with restoration of ciliated pseudostratified columnar epithelium, preservation of the basal cell layer, and reduction of inflammatory infiltrates compared with immediate post-dilation specimens. In one ET, the area corresponding to a previously crushed lymphoid follicle demonstrated replacement by a thin fibrous layer without evidence of excessive fibrosis or progressive scarring. Although these observations were derived from a single pediatric patient, they provide limited descriptive histological observations suggesting a possible healing-related tissue response following dilation. Similar postoperative histological observations have also been reported in adult cohorts following balloon dilation using dedicated Eustachian tuboplasty systems^11^.

Experimental models have similarly demonstrated that balloon dilation may induce acute mucosal injury-related changes followed by subsequent epithelial healing-related alterations. In a rat model, epithelial desquamation and cartilage microfracture were followed by reappearance of ciliated epithelium and partial remodeling at 12 weeks^23^. Similarly, Jia et al. demonstrated in a minipig model that balloon expansion induces mechanical mucosal compression, focal epithelial stripping, and transient ischemic changes within the tubal wall, followed by progressive epithelial regeneration and restoration of pseudostratified ciliated epithelium^24^. The similarities between these experimental observations and the present human findings support the biological plausibility that balloon dilation induces acute mechanical mucosal injury followed by subsequent healing-related epithelial changes. However, definitive conclusions regarding long-term remodeling mechanisms or therapeutic pathways cannot be established from the present study.

Exploratory subgroup analysis according to 6-month tympanometric outcome did not establish statistically significant clinicopathological associations. However, descriptive numerical differences between improved and non-improved ears may reflect relatively greater preservation of basal epithelial integrity in ears demonstrating favorable tympanometric improvement. These observations should be interpreted cautiously, given the limited sample size, the exploratory nature of the subgroup analysis, and the fact that immediate post-dilation biopsies primarily reflect acute mechanical tissue effects rather than long-term healing-related remodeling. In addition, long-term follow-up histopathological assessment was available from only two ETs in a single patient, limiting meaningful clinicopathological correlation.

Immediate post-dilation findings, including epithelial disruption and lymphoid tissue compression reflected by crushing artifacts, were consistent with acute mechanical tissue effects of balloon dilation. Preservation of the basal epithelial layer in the majority of specimens may indicate maintenance of the regenerative epithelial compartment and a potential for subsequent epithelial repair. Collectively, these observations may provide preliminary insight into the early tissue responses following balloon dilation and their possible relationship to the favorable functional outcomes observed in our previously reported cohort^9^.

The functional outcomes observed in this cohort are consistent with previous pediatric studies of BET, which have reported tympanometric normalization rates ranging from approximately 39% to over 80% across varying follow-up intervals^15,25–27^. While the present study primarily focused on histopathological outcomes, the observed functional improvement may provide a supportive clinical context for the observed tissue-level findings. However, the present study was not designed to establish direct clinicopathological correlations.

The present findings also align with emerging clinical data describing the use of standard endovascular balloons for BET. Ungar et al. reported clinical improvement and procedural application of endovascular balloons, suggesting their potential as a practical and cost-effective alternative to proprietary systems^28^. Although histopathological data were not included in that study, the current findings suggest that endovascular balloons may induce mucosal tissue response patterns broadly comparable to those previously reported with dedicated balloon dilation devices. However, the present study was not designed to establish mechanistic conclusions regarding therapeutic efficacy, reparative tissue responses, or the relative biological effects of different balloon systems.

Several limitations should be considered. The sample size was relatively small, with histopathological analysis performed on 22 ETs from 11 pediatric patients. Long-term histological follow-up was limited to two ETs from a single patient at 16 weeks. Additional postoperative biopsies were not performed due to ethical constraints associated with repeated invasive sampling in pediatric patients without a clinical indication. Similar limitations have been reported in previous human histopathological studies, including that of Kivekäs et al., in which follow-up biopsies were obtained in only a small subset of patients^11^.

Furthermore, histological sampling was limited to the posterior cushion of the ET at the pharyngeal orifice and therefore may not fully represent the entire mucosal circumference of the ET, particularly regions such as the luminal floor that may experience different degrees of mechanical contact during balloon dilation. However, this biopsy location was intentionally selected to provide safe, reproducible, and anatomically consistent sampling across all cases, consistent with previously published histopathological studies of BET^11^. This study was not designed to establish direct correlations between histopathological findings and clinical outcomes, as clinical endpoints were analyzed separately. Despite these limitations, this study provides paired pre- and post-dilation histopathological data in a pediatric population and offers novel insight into mucosal responses following balloon dilation using a standard endovascular balloon catheter. By extending existing evidence beyond adult cohorts and experimental models, the present findings may provide preliminary histopathological insight into healing-related mucosal changes following balloon dilation.

This study provides histopathological insight into the mucosal response of the pediatric ET following balloon dilation using a standard endovascular balloon. The observed pattern of acute superficial epithelial injury with preservation of the basal epithelial layer may reflect maintenance of the regenerative epithelial compartment and a potential for subsequent epithelial repair. Limited follow-up histological observations from one patient demonstrated partial re-epithelialization with reduction in inflammatory infiltrates; however, these findings remain preliminary and hypothesis-generating and do not permit definitive conclusions regarding reparative response or tissue remodeling following dilation.

Collectively, the present findings provide preliminary histopathological observations regarding mucosal responses following balloon dilation using a standard endovascular balloon in pediatric obstructive ETD. However, larger prospective studies with extended histological and clinical follow-up are required before definitive conclusions regarding long-term biological effects, safety, feasibility, or broader clinical applicability can be established.

## Supplementary Information

Below is the link to the electronic supplementary material.

Supplementary Material 1

Supplementary Material 2

## Data Availability

The datasets generated and analyzed during the current study are available from the corresponding author on reasonable request.
