# Supplementary material for: Histopathological changes of the Eustachian tube mucosa following balloon Eustachian tuboplasty in children: insights from standard endovascular balloon application
Source: Sci Rep. 2026 Jul 21;16:22821. doi: 10.1038/s41598-026-57118-9 (PMC13389457; doi:10.1038/s41598-026-57118-9)
Supplement: Supplementary file 1 — Supplementary Material 1 [file 41598_2026_57118_MOESM1_ESM.docx]

**Histopathological changes of the Eustachian tube mucosa following balloon Eustachian tuboplasty in children: insights from standard endovascular balloon application**

**Short running title:** Histopathological Changes After Balloon Eustachian Tuboplasty

**Abdelrahman Mostafa Hassan^1*^, Hani Farouk Elgarem^2^, Eman Sheta^3^,**

**Yasser Gaber Sheweal^2^, Yehia Mohammed Ashry^4^**

1. Assistant Lecturer of Otorhinolaryngology, Faculty of Medicine, Alexandria University, Alexandria, Egypt.

2. Professor of Otorhinolaryngology, Faculty of Medicine, Alexandria University, Alexandria, Egypt.

3. Assistant Professor of Pathology, Faculty of Medicine, Alexandria University, Alexandria, Egypt.

4. Lecturer of Otorhinolaryngology, Faculty of Medicine, Suez Canal University, Suez Canal, Egypt.

*** Corresponding author**

**Name: Abdelrahman Mostafa Hassan**

**Postal codes:** 21611

**Telephone:** +20 155 4517557

**e-mail:** a_hassan181@alexmed.edu.eg

**Supplementary Table S1. Inter-observer agreement assessed using intraclass correlation coefficients (ICC) and Cohen’s weighted kappa for mucosal and submucosal histopathological parameters (n = 44).**

|  | **ICC (LL – UL 95%C.I)** | **κ (95% C.I)** |
| --- | --- | --- |
| Epithelium quality | 0.958 (0.923 – 0.977) | 0.920 (0.869 – 0.970) |
| Cilia | 0.933 (0.877 – 0.964) | 0.874 (0.761 – 0.987) |
| Squamous metaplasia | 1.000 (1.000 – 1.000) | 1.000 (1.000 – 1.000) |
| Intraepithelial inflammation | 0.901 (0.819 – 0.946) | 0.805 (0.627 – 0.984) |
| Basal cell integrity | 0.957 (0.921 – 0.977) | 0.917 (0.846 – 0.988) |
| Submucosal inflammation | 0.923 (0.860 – 0.958) | 0.856 (0.763 – 0.949) |
| Lymphoid follicles | 1.000 (1.000 – 1.000) | 1.000 (1.000 – 1.000) |
| Quantity of fibrosis | 0.926 (0.865 – 0.960) | 0.863 (0.724 – 1.000) |
| Submucosal glands | 1.000 (1.000 – 1.000) | 1.000 (1.000 – 1.000) |
| Crushing artifact | 0.951 (0.910 – 0.973) | 0.906 (0.779 – 1.000) |

ICC: Intraclass correlation coefficient; κ: Cohen’s weighted kappa coefficient with quadratic weighting; CI: confidence interval; LL: lower limit; UL: upper limit.

| **Value of ICC** | **Strength of agreement^#^** |
| --- | --- |
| Below 0.50 | Poor |
| 0.50 and <0.75 | Moderate |
| 0.75 and 0.90 | Good |
| Above 0.90 | Excellent |

**#: By Koo and Li (2016)**

| **Value of κ** | **Strength of agreement** |
| --- | --- |
| <0.00 | Poor agreement |
| 0.00 – 0.20 | Slight agreement |
| 0.21 – 0.40 | Fair agreement |
| 0.41 – 0.60 | Moderate agreement |
| 0.61 – 0.80 | Good agreement |
| 0.81 – 1.00 | Very good agreement |

**Supplementary Table S2.Descriptive paired distribution of mucosal histopathological parameters before and after balloon dilation (n = 22 Eustachian tubes).**

|  | **Before** | **After** | **Test of Sig (p)** | **Effect Size (95% C.I)** |
| --- | --- | --- | --- | --- |
| **Epithelium quality** |  |  |  |  |
| Ulcerated | 4 (18.2%) | 16 (72.7%) | MH=16.500^*^ p=0.001^*^ | Cramer's V= 0.255 (0.209 – 0.615) |
| Fair | 5 (22.7%) | 3 (13.6%) |  |  |
| Good | 10 (45.5%) | 3 (13.6%) |  |  |
| Excellent | 3 (13.6%) | 0 (0.0%) |  |  |
| Mean ± SD. | 1.55 ± 0.96 | 0.41 ± 0.73 | Z=3.206^*^ p=0.001^*^ | r=0.683 (0.368 – 0.858) |
| Median (IQR) | 2.0 (1.0 – 2.0) | 0.0 (0.0 – 1.0) |  |  |
| **Cilia** |  |  |  |  |
| Absent | 8 (36.4%) | 16 (72.7%) | MH=9.000^*^ p=0.001^*^ | Cramer's V= 0.589 (0.340 – 0.890) |
| Fair | 10 (45.5%) | 6 (27.3%) |  |  |
| Present | 4 (18.2%) | 0 (0.0%) |  |  |
| Mean ± SD. | 0.82 ± 0.73 | 0.27 ± 0.46 | Z=3.207^*^ p=0.001^*^ | r=0.684 (0.368 – 0.858) |
| Median (IQR) | 1.0 (0.0 – 1.0) | 0.0 (0.0 – 1.0) |  |  |
| **Squamous metaplasia** |  |  |  |  |
| Absent | 20 (90.9%) | 20 (90.9%) | McN=0.000 p=1.000 | φ=0.100 (0.048 – 0.222) |
| Present | 2 (9.1%) | 2 (9.1%) |  |  |
| Mean ± SD. | 0.09 ± 0.29 | 0.09 ± 0.29 | Z=0.000 p=1.000 | r=0.000 (-0.422 – 0.422) |
| Median (IQR) | 0.0 (0.0 – 0.0) | 0.0 (0.0 – 0.0) |  |  |
| **Intraepithelial inflammation** |  |  |  |  |
| Absent | 11 (50.0%) | 19 (86.4%) | McN=6.705 p=0.008^*^ | φ=0.397 (0.199 – 0.652) |
| Present | 11 (50.0%) | 3 (13.6%) |  |  |
| Mean ± SD. | 0.50 ± 0.51 | 0.14 ± 0.35 | Z=2.828^*^ p=0.005^*^ | r=0.603 (0.243 – 0.817) |
| Median (IQR) | 0.50 (0.0 – 1.0) | 0.0 (0.0 – 0.0) |  |  |
| **Basal cell integrity** |  |  |  |  |
| Ulcerated | 0 (0.0%) | 2 (9.1%) | MH=21.000 p=0.201 | Cramer's V=0.420 (0.345 – 0.726) |
| Fair | 5 (22.7%) | 4 (18.2%) |  |  |
| Good | 7 (31.8%) | 9 (40.9%) |  |  |
| Excellent | 10 (45.5%) | 7 (31.8%) |  |  |
| Mean ± SD. | 2.23 ± 0.81 | 1.95 ± 0.95 | Z=1.231 p=0.218 | r=0.262 (-0.179 – 0.616) |
| Median (IQR) | 2.0 (2.0 – 3.0) | 2.0 (1.0 – 3.0) |  |  |

IQR: interquartile range; SD: standard deviation; Z: Wilcoxon signed-rank test; McN: McNemar test; MH: Marginal Homogeneity test.
p: p-value for comparison between pre- and post-dilation findings.
*Statistically significant at p ≤ 0.05.

**Supplementary Table S3. Descriptive paired distribution of submucosal histopathological parameters before and after balloon dilation (n = 22 Eustachian tubes).**

|  | **Before** | **After** | **Test of Sig (p)** | **Effect Size (95% C.I)** |
| --- | --- | --- | --- | --- |
| **SM inflammation** |  |  |  |  |
| None | 0 (0.0%) | 1 (4.5%) | MH=22.000 p=0.346 | Cramer's V=0.425 (0.306 – 0.680) |
| Mild | 3 (13.6%) | 8 (36.4%) |  |  |
| Moderate | 13 (59.1%) | 4 (18.2%) |  |  |
| Severe | 6 (27.3%) | 9 (40.9%) |  |  |
| Mean ± SD. | 2.14 ± 0.64 | 1.95 ± 1.0 | Z=0.966 p=0.334 | r=0.206 (-0.236 – 0.577) |
| Median (IQR) | 2.0 (2.0 – 3.0) | 2.0 (1.0 – 3.0) |  |  |
| **Lymphoid follicles** |  |  |  |  |
| Absent | 18 (81.8%) | 21 (95.5%) | McN=2.031 p=0.250 | φ=0.463 (0.319 – 1.000) |
| Present | 4 (18.2%) | 1 (4.5%) |  |  |
| Mean ± SD. | 0.18 ± 0.39 | 0.05 ± 0.21 | Z=1.732 p=0.083 | r=0.369 (-0.062 – 0.684) |
| Median (IQR) | 0.0 (0.0 – 0.0) | 0.0 (0.0 – 0.0) |  |  |
| **Quantity of fibrosis** |  |  |  |  |
| None | 0 (0.0%) | 2 (9.1%) | MH=12.000 p=0.317 | Cramer's V=0.247 (0.134 – 0.704) |
| Mild | 2 (9.1%) | 12 (54.5%) |  |  |
| Moderate | 16 (72.7%) | 8 (36.4%) |  |  |
| Severe | 4 (18.2%) | 0 (0.0%) |  |  |
| Mean ± SD. | 1.09 ± 0.53 | 1.27 ± 0.63 | Z=0.973 p=0.331 | r=0.207 (-0.235 – 0.578) |
| Median (IQR) | 1.0 (1.0 – 1.0) | 1.0 (1.0 – 2.0) |  |  |
| **SM Glands** |  |  |  |  |
| None present | 12 (54.5%) | 4 (18.2%) | MH=27.000 p=0.055 | Cramer's V=0.268 (0.150 – 0.551) |
| Fair | 1 (4.5%) | 0 (0.0%) |  |  |
| Good | 1 (4.5%) | 9 (40.9%) |  |  |
| Excellent | 8 (36.4%) | 9 (40.9%) |  |  |
| Mean ± SD. | 1.23 ± 1.45 | 2.05 ± 1.09 | Z=1.896 p=0.058 | r=0.404 (-0.021 – 0.706) |
| Median (IQR) | 0.0 (0.0 – 3.0) | 2.0 (2.0 – 3.0) |  |  |
| **Crushing** |  |  |  |  |
| Absent | 20 (90.9%) | 6 (27.3%) | McN=18.427 p=0.001^*^ | φ=0.161 (0.025 – 0.642) |
| Marked | 2 (9.1%) | 16 (72.7%) |  |  |

IQR: **Inter quartile range** SD: **Standard deviation** Z: **Wilcoxon signed ranks test**

**McN: McNemar test MH: Marginal Homogeneity Test**

**SM: submucosa**

p: p value for comparing between **before** and **after**

*: Statistically significant at p ≤ 0.05

**Supplementary Table S4. Descriptive subgroup distribution of mucosal histopathological parameters according to 6-month tympanometric outcome.(n = 22 Eustachian tubes).**

|  |  | **Clinical improvement** | | | |  |  |
| --- | --- | --- | --- | --- | --- | --- | --- |
|  | **Mucosa** | **Improved (n = 17)** | | **Not improved (n = 5)** | | **χ^2^** | **p** |
|  |  | **No.** | **%** | **No.** | **%** |  |  |
| **Epithelium quality** | **Before** |  |  |  |  |  |  |
|  | Ulcerated (0) | 2 | 11.8 | 2 | 40.0 | 2.340 | ^MC^p= 0.618 |
|  | Fair (1) | 4 | 23.5 | 1 | 20.0 |  |  |
|  | Good (2) | 8 | 47.1 | 2 | 40.0 |  |  |
|  | Excellent (3) | 3 | 17.6 | 0 | 0.0 |  |  |
|  | **After** |  |  |  |  |  |  |
|  | Ulcerated (0) | 11 | 64.7 | 5 | 100.0 | 1.517 | ^MC^p= 0.399 |
|  | Fair (1) | 3 | 17.6 | 0 | 0.0 |  |  |
|  | Good (2) | 3 | 17.6 | 0 | 0.0 |  |  |
|  | Excellent (3) | 0 | 0.0 | 0 | 0.0 |  |  |
| **Cilia** | **Before** |  |  |  |  |  |  |
|  | Absent | 5 | 29.4 | 3 | 60.0 | 2.055 | ^MC^p= 0.443 |
|  | Fair | 9 | 52.9 | 1 | 20.0 |  |  |
|  | Preserved | 3 | 17.6 | 1 | 20.0 |  |  |
|  | **After** |  |  |  |  |  |  |
|  | Absent | 13 | 76.5 | 3 | 60.0 | 0.528 | ^MC^p= 0.585 |
|  | Fair | 4 | 23.5 | 2 | 40.0 |  |  |
|  | Preserved | 0 | 0.0 | 0 | 0.0 |  |  |
| **Squamous metaplasia** | **Before** |  |  |  |  |  |  |
|  | Absent | 15 | 88.2 | 5 | 100.0 | 0.647 | ^FE^p= 1.000 |
|  | Present | 2 | 11.8 | 0 | 0.0 |  |  |
|  | **After** |  |  |  |  |  |  |
|  | Absent | 15 | 88.2 | 5 | 100.0 | 0.647 | ^FE^p= 1.000 |
|  | Present | 2 | 11.8 | 0 | 0.0 |  |  |
| **Intra epithelial inflammation** | **Before** |  |  |  |  |  |  |
|  | Absent | 8 | 47.1 | 3 | 60.0 | 0.259 | ^FE^p= 1.000 |
|  | Present | 9 | 52.9 | 2 | 40.0 |  |  |
|  | **After** |  |  |  |  |  |  |
|  | Absent | 14 | 82.4 | 5 | 100.0 | 1.022 | ^FE^p= 1.000 |
|  | Present | 3 | 17.6 | 0 | 0.0 |  |  |
| **Basal cell integrity** | **Before** |  |  |  |  |  |  |
|  | Ulcerated (0) | 0 | 0.0 | 0 | 0.0 | 2.094 | ^MC^p= 0.442 |
|  | Fair (1) | 3 | 17.6 | 2 | 40.0 |  |  |
|  | Good (2) | 5 | 29.4 | 2 | 40.0 |  |  |
|  | Excellent (3) | 9 | 52.9 | 1 | 20.0 |  |  |
|  | **After** |  |  |  |  |  |  |
|  | Ulcerated (0) | 2 | 11.8 | 0 | 0.0 | 4.485 | ^MC^p= 0.185 |
|  | Fair (1) | 2 | 11.8 | 2 | 40.0 |  |  |
|  | Good (2) | 6 | 35.3 | 3 | 60.0 |  |  |
|  | Excellent (3) | 7 | 41.2 | 0 | 0.0 |  |  |

χ²: Chi-square test; FET: Fisher’s exact test; MC: Monte Carlo test.
p: p-value for comparison between the studied groups.

**Supplementary Table S5.Descriptive subgroup distribution of submucosal histopathological parameters according to 6-month tympanometric outcome.(n = 22 Eustachian tubes).**

|  |  | **Clinical improvement** | | | |  |  |
| --- | --- | --- | --- | --- | --- | --- | --- |
|  | **Submucosal parameters** | **Improved (n = 17)** | | **Not improved(n = 5)** | | **χ^2^** | **p** |
|  |  | **No.** | **%** | **No.** | **%** |  |  |
| **Submucosal inflammation** | **Before** |  |  |  |  |  |  |
|  | None (0) | 0 | 0.0 | 0 | 0.0 | 1.068 | ^MC^p= 0.806 |
|  | Mild (1) | 3 | 17.6 | 0 | 0.0 |  |  |
|  | Moderate (2) | 10 | 58.8 | 3 | 60.0 |  |  |
|  | Severe (3) | 4 | 23.5 | 2 | 40.0 |  |  |
|  | **After** |  |  |  |  |  |  |
|  | None (0) | 1 | 5.9 | 0 | 0.0 | 2.272 | ^MC^p= 0.657 |
|  | Mild (1) | 5 | 29.4 | 3 | 60.0 |  |  |
|  | Moderate (2) | 4 | 23.5 | 0 | 0.0 |  |  |
|  | Severe (3) | 7 | 41.2 | 2 | 40.0 |  |  |
| **Lymphoid follicles** | **Before** |  |  |  |  |  |  |
|  | Absent (0) | 14 | 82.4 | 4 | 80.0 | 0.014 | ^FE^p= 1.000 |
|  | Present (1) | 3 | 17.6 | 1 | 20.0 |  |  |
|  | **After** |  |  |  |  |  |  |
|  | Absent (0) | 16 | 94.1 | 5 | 100.0 | 0.308 | ^FE^p= 1.000 |
|  | Present (1) | 1 | 5.9 | 0 | 0.0 |  |  |
| **Quantity of fibrosis** | **Before** |  |  |  |  |  |  |
|  | None (0) | 2 | 11.8 | 0 | 0.0 | 1.635 | ^MC^p= 0.549 |
|  | Mild (1) | 11 | 64.7 | 5 | 100.0 |  |  |
|  | Moderate (2) | 4 | 23.5 | 0 | 0.0 |  |  |
|  | Severe (3) | 0 | 0.0 | 0 | 0.0 |  |  |
|  | **After** |  |  |  |  |  |  |
|  | None (0) | 1 | 5.9 | 1 | 20.0 | 1.564 | ^MC^p= 0.618 |
|  | Mild (1) | 10 | 58.8 | 2 | 40.0 |  |  |
|  | Moderate (2) | 6 | 35.3 | 2 | 40.0 |  |  |
|  | Severe (3) | 0 | 0.0 | 0 | 0.0 |  |  |
| **Submucosal glands** | **Before** |  |  |  |  |  |  |
|  | Not identified (0) | 9 | 52.9 | 3 | 60.0 | 1.137 | ^MC^p= 1.000 |
|  | Fair (1) | 1 | 5.9 | 0 | .0 |  |  |
|  | Good (2) | 1 | 5.9 | 0 | .0 |  |  |
|  | Excellent (3) | 6 | 35.3 | 2 | 40.0 |  |  |
|  | **After** |  |  |  |  |  |  |
|  | Not identified (0) | 3 | 17.6 | 1 | 20.0 | 0.364 | ^MC^p= 1.000 |
|  | Fair (1) | 0 | 0.0 | 0 | 0.0 |  |  |
|  | Good (2) | 7 | 41.2 | 2 | 40.0 |  |  |
|  | Excellent (3) | 7 | 41.2 | 2 | 40.0 |  |  |

χ²: Chi-square test; FET: Fisher’s exact test; MC: Monte Carlo test.
p: p-value for comparison between the studied groups.
